# 2381: Characterizing delayed transition to adult care in children with chronic kidney disease

**DOI:** 10.1017/cts.2017.267

**Published:** 2018-05-10

**Authors:** Sarahfaye Dolman, Richard Caplan, Mitchell R. Fawcett, Edward Ewen, Joshua Zaritsky, H. Timothy Bunnell, Rubeen Israni, Sidney J. Swanson, Claudine Jurkovitz

**Affiliations:** Christiana Care Health System, Value Institute, Newark, DE, USA

## Abstract

OBJECTIVES/SPECIFIC AIMS: As part of a larger effort to create a longitudinal record of care for patients with chronic kidney disease (CKD) in Delaware, we assessed transitions of care from pediatric to adult care. This study examined the length of time between last pediatric contact and first contact in the adult system in order to determine characteristics associated with delayed transition to adult care. METHODS/STUDY POPULATION: Patients who receive pediatric care at the Nemours/Alfred I. duPont Hospital for Children (Nemours) are transitioned to adult care between the ages of 18 and 21. Our study population consists of all patients seen in the Nephrology unit at Nemours for CKD, hypertension (HTN), or diabetes who turned 21 years old between 2007 and 2013. Records of office visits from Nemours, Christiana Care Health System (CCHS), and Nephrology Associates, P.A. (NAPA) were transformed into the OMOP common data model and merged. Patients who had at least 1 record in the Nemours EHR of pediatric care before age 21 and had at least 1 record in the CCHS or NAPA adult EHRs were considered transitioned. To identify characteristics associated with delayed transition to adult care, we compared gender, race, ethnicity, age, comorbidities, and level of kidney function at the last pediatric visit between patients whose transition gap was less than 1 year and patients whose gap was 1 year or more. Kidney function was estimated by calculating glomerular filtration rate (GFR). Nemours estimates GFR in children using the revised Schwartz equation, which is based on serum creatinine and height. To calculate adult GFR, we used the CKD-Epi equation, which is based on serum creatinine, age, sex, and race and is widely used to derive adult GFR. As kidney function declines, GFR decreases. We used Fisher exact test to compare categorical variables and *t*-test to compare age and GFR. RESULTS/ANTICIPATED RESULTS: We found only 109 (25%) patients who had records in our adult offices out of the 440 Nemours patients in our data set. Of the 109 transitioned patients, 54 had office visits at CCHS, 37 at NAPA, and 18 at both locations. Examining the office visits of the 109 transitioned patients, 34 (31%) had an overlap in visits defined as an office visit at CCHS or NAPA before the last office visit at Nemours, and 75 (69%) did not have an overlap. The median gap between last pediatric and first adult office visit for the 75 patients without an overlap was 615 days (range 8–3495 d). Only 6 (6%) of the 109 transitioned patients had overlapping GFR measurements from pediatric to adult care, and all of the adult GFR calculations (CKD-Epi) were greater than the pediatric GFR calculations (Schwartz). The difference between child and adult GFR ranged from 8.2 to 87.1 mL/minute per 1.72 m^2^. DISCUSSION/SIGNIFICANCE OF IMPACT: During the transition from pediatric care to adult care, many young adults with CKD experience declines in health outcomes and comorbidities such as diabetes and HTN complicate self-management. Lack of overlap between pediatric and adult care office visits indicates a delay in executing this transition. In our population of 109 transitioned patients, 69% did not have an overlap in care, and 50% of those without overlap had a gap of more than 615 days (1 y, 8 mo). Our analysis suggests that young adults who are younger at last pediatric office visit are more likely to delay transitioning to adult care. Transitioning from the nurturing environment of pediatric care to adult care is a complex process and could be challenging for young adults with CKD. Transition clinics may be necessary to improve the coordination of care and help these young adults keep their physician appointments.

